# Knowledge, attitude and practice of patients with immune-mediated and inflammatory rheumatic diseases towards mental health and psychological interventions

**DOI:** 10.3389/fpubh.2026.1959054

**Published:** 2026-10-21

**Authors:** Shanshan Li, Xue Wang, Hong Liu, Minghuan Liu, Ying Liu, Yanan Sun, Dongxia Tao, Bailing Tian, Fang Wang

**Affiliations:** 1 Department of Otolaryngology, The First Hospital of China Medical University, Shenyang, China; 2 Department of Rheumatology and Immunology, The First Hospital of China Medical University, Shenyang, China; 3 Department of Day surgery Ward, The First Hospital of China Medical University, Shenyang, China; 4 Department of Nephrology, The First Hospital of China Medical University, Shenyang, China; 5 Department of Nursing, The First Hospital of China Medical University, Shenyang, China

**Keywords:** attitude, cross-sectional study, knowledge, mental health, practice, rheumatoid

## Abstract

**Introduction:**

Autoimmune rheumatologic diseases are chronic, recurrent conditions that often cause pain, disability, and high treatment costs. These disease burdens increase the risk of anxiety, depression, and sleep problems, yet many patients do not receive timely psychological support. Understanding how patients perceive mental health and psychological interventions, and how they act on these perceptions, is important for designing effective education and support strategies.

**Methods:**

This multicenter cross-sectional study was conducted in six hospitals in China between July and December 2023 and patients with immune-mediated and inflammatory rheumatic diseases were included. Demographic information and KAP scores were obtained using a self-designed questionnaire.

**Results:**

A total of 672 valid questionnaires were collected, with 499 female respondents and a mean age of 44.86 ± 15.54 years. The mean KAP scores were 5.65 ± 4.812 (range: 0–18), 23.03 ± 4.125 (range: 6–30), and 18.41 ± 4.179 (range: 6–30). Multivariable logistic regression with an *a priori* confounder selection strategy showed that knowledge (OR = 1.133, 95% CI: 1.086–1.182, *p* < 0.001), attitude (OR = 1.115, 95% CI: 1.059–1.173, *p* < 0.001), and age (OR = 1.023, 95% CI: 1.008–1.037, *p* = 0.002) were independently associated with practice. Patients with immune-mediated and inflammatory rheumatic diseases demonstrated inadequate knowledge, positive attitudes, and inactive practice towards mental health and psychological interventions. Structural equation modeling indicated that knowledge was directly associated with attitude (*β* = 0.369, *p* = 0.001) and practice (*β* = 0.518, *p* = 0.001), with attitude also showing a direct path to practice (*β* = 0.202, *p* = 0.001).

**Conclusion:**

Patients with immune-mediated and inflammatory rheumatic diseases exhibit notable gaps in mental health knowledge, attitudes, and practices. These findings provide valuable empirical targets for future prospective research and highlight the potential need for tailored educational programs to support patient wellbeing.

## Introduction

Immune-mediated and inflammatory rheumatic diseases are a diverse group of disorders that mainly affect joints and connective tissues (1). In China, they are important causes of chronic morbidity because they often involve multiple tissues and organs and can lead to progressive disability (2, 3). Common conditions include rheumatoid arthritis, systemic lupus erythematosus, Sjögren’s syndrome, systemic sclerosis, inflammatory myopathies, vasculitis, ankylosing spondylitis, and gout (4–6). Many patients experience recurrent flares, long disease duration, functional limitations, and high treatment costs, which together place a heavy physical, social, and economic burden on patients and families (5, 6).

These long-term burdens increase the risk of mental health problems. Patients with rheumatic diseases have high rates of anxiety, depression, sleep disturbance, fatigue, and psychological distress (7–9). These problems can worsen pain and disease activity, reduce adherence, and impair quality of life (10–12). However, many patients do not receive timely psychological support or formal mental health care (7). In routine practice, rheumatology services still focus mainly on physical symptoms, while patients’ understanding and use of mental health and psychological interventions remain unclear.

In this study, we use the term *mental health* to refer to common psychological problems in this population, including anxiety, depressive symptoms, and sleep disturbance (8, 9, 13). We use the term *psychological interventions* to include non-pharmacological and psychosocial approaches such as health education, psychological counseling, psychotherapy (e.g., cognitive behavioral therapy), and structured mental health support delivered by psychologists, psychiatrists, or trained nurses (7, 14, 15). Understanding how patients think about these issues and what they actually do in daily life is important for designing effective support strategies.

The knowledge, attitude, and practice (KAP) model is a structured approach to assess what people know about a health topic, how they feel about it, and how they behave in relation to it (14, 15). In patients with chronic diseases, KAP surveys have been used to identify gaps in awareness, misconceptions, and barriers to healthy behavior, and to guide targeted education and self-management programs (16, 17). In rheumatology, previous studies have described the prevalence and impact of depression, anxiety, and sleep problems (8, 9), but few have systematically examined patients’ KAP regarding mental health and psychological interventions across different autoimmune rheumatologic diseases.

To address this gap, we conducted a multicenter crosssectional study in patients with immune-mediated and inflammatory rheumatic diseases (including rheumatoid arthritis, systemic lupus erythematosus, Sjögren’s syndrome, systemic sclerosis, inflammatory myopathy, vasculitis, ankylosing spondylitis, gout, and other connective tissue diseases) attending rheumatology departments in six tertiary hospitals in China. Using a self-designed KAP questionnaire and validated scales for anxiety, depression, and sleep, we aimed to describe patients’ knowledge, attitudes, and practices related to mental health and psychological interventions; examine the associations between KAP scores and demographic and clinical characteristics; and explore the relationships among knowledge, attitude, and practice using correlation analysis and structural equation modeling. These objectives were based on the hypothesis that higher levels of knowledge would be associated with more positive attitudes and more active practice, and that better KAP would be linked to lower levels of anxiety, depression, and sleep disturbance.

## Materials and methods

### Study design and participants

This was a multicenter cross-sectional study conducted in the rheumatology and immunology departments of six tertiary A hospitals in China between July and December 2023 (Supplementary Table 1). The study followed the STROBE recommendations for reporting observational studies.

We included adult patients with immune-mediated and inflammatory rheumatic diseases who attended the rheumatology outpatient clinics or inpatient wards during the study period.

*Inclusion criteria were*: 1. Age ≥ 18 years; 2. Diagnosis of an autoimmune rheumatologic disease (e.g., rheumatoid arthritis, systemic lupus erythematosus, Sjögren’s syndrome, systemic sclerosis, inflammatory myopathy, vasculitis, ankylosing spondylitis, gout, and other connective tissue diseases) documented by a rheumatologist in the medical record according to current classification criteria, or highly suspected clinical cases actively undergoing final diagnostic confirmation (categorized as “undifferentiated/unclassified rheumatic diseases”); 3. Able to communicate in Chinese and complete the questionnaire independently or with minimal assistance; and 4. Provided written informed consent.

*Exclusion criteria were*: 1. Presence of serious organic diseases judged likely to interfere with participation or to confound mental health assessment (e.g., advanced malignancy, decompensated heart failure, end-stage renal disease, severe chronic liver disease, major neurological disorders such as stroke with marked cognitive impairment); 2. Primary non-rheumatologic conditions as the main reason for current admission or visit; 3. Documented severe psychiatric disorders (e.g., schizophrenia, bipolar disorder, severe cognitive impairment) that prevented reliable completion of the questionnaire; and 4. Refusal or inability to provide informed consent. We did not exclude patients solely on the basis of common mental health conditions such as depression or anxiety, as these were key outcomes in this study.

### Case identification

Potential participants were identified by trained research nurses and physicians using the daily clinic and ward lists. Diagnoses were confirmed through review of electronic medical records and rheumatologist documentation. Where applicable, standard diagnostic terms and corresponding ICD-10 codes were used (e.g., M05–M06 for rheumatoid arthritis, M32 for systemic lupus erythematosus, M35.0 for Sjögren’s syndrome).

The protocol was approved by the Medical Ethics Committee of authors’ hospital. The same written consent form and procedure were used in all six hospitals. Trained research staff explained the study, answered questions, and obtained written informed consent from all participants before questionnaire administration, in accordance with the Declaration of Helsinki.

### Procedures

We used stratified sampling at the hospital level and convenience sampling within each hospital. The six tertiary hospitals were selected to represent different regions and large rheumatology centers. Within each rheumatology/immunology department, all eligible patients attending outpatient clinics or admitted to wards during data-collection periods were consecutively invited to participate until the target sample size for that site was reached. Recruitment was performed on different days and at different times to reduce selection bias.

The KAP questionnaire was developed in Chinese with reference to relevant guidelines and literature (10–12, 18). An initial item pool was drafted by rheumatologists, psychologists, and nurses, then reviewed by 10 experts in rheumatology, immunology, psychological medicine, and nursing. Two rounds of expert consultation were used to assess importance, relevance, and clarity. Items with low importance or high variation were revised or removed. For example, the first knowledge item was revised to “I understand what psychological intervention is. (Psychological intervention refers to a process guided by psychological theories, systematically influencing certain individuals’ psychological activities, personality traits, or issues towards the expected goal changes.)”. A pilot study with 31 patients assessed initial feasibility, and minor wording changes were made based on feedback. The psychometric properties of the final questionnaire were comprehensively evaluated using the full analytical sample (*N* = 672). In our sample, the questionnaire demonstrated excellent internal consistency, with an overall Cronbach’s α of 0.904. The separate Cronbach’s α coefficients for the knowledge, attitude, and practice subscales were 0.938, 0.920, and 0.769, respectively. Construct validity was evaluated via Confirmatory Factor Analysis (CFA) using AMOS 26.0 with maximum likelihood estimation. The CFA confirmed the theoretical three-factor structure with good model fit indices: CMIN/DF = 3.227, RMSEA = 0.058, IFI = 0.965, TLI = 0.956, and CFI = 0.964. All standardized factor loadings were statistically significant (*p* < 0.001) (Supplementary Figure S1).

The final self-administered questionnaire (in Chinese) comprised four parts (Details are provided in the questionnaire file). Part I collected demographic and clinical information, including age, sex, residence, education, occupation, monthly household income per capita, marital status, smoking and drinking habits, type and duration of rheumatic disease, family history of rheumatic disease and mental illness, presence of mental illness, and type of health insurance. Part II (knowledge) included nine items scored 2 (“very well known”), 1 (“somewhat known”), or 0 (“unknown”), for a total score of 0–18. Part III (attitude) comprised six items scored on a 5-point Likert scale from 5 (“strongly agree”) to 1 (“strongly disagree”), with a total score of 6–30. Part IV (practice) included six items scored from 5 (“always”) to 1 (“never”), also with a total score of 6–30. Consistent with previous KAP studies (18), a score >70% of the maximum in each domain (> 12.6 for knowledge, > 21 for attitude, > 21 for practice) was defined as adequate knowledge, positive attitude, or proactive practice. All response options are presented as numbered Likert scales in both the main text and Supplementary Questionnaire file.

Depressive symptoms, anxiety symptoms, and sleep quality were screened and assessed using the Self-Rating Depression Scale (SDS), the Self-Rating Scale for Anxiety (SAS), and the Pittsburgh Sleep Quality Index (PSQI). These instruments are validated screening tools rather than diagnostic instruments for formal psychiatric disorders. Validated Chinese normative cutoffs and references were applied where symptom severity categories were referenced (13, 19). The SDS contains 20 items, each scored from 1 (“never or occasionally”) to 4 (“always”), with total scores ranging from 20 to 80; higher scores indicate more severe depressive symptoms. The SAS has 20 items rated from 1 (“not or rarely present”) to 4 (“present most or all of the time”); 15 items are scored directly and 5 positively worded items (items 5, 9, 13, 17, 19) are reverse-scored. The PSQI assesses sleep quality over the past month using 19 self-rated items, from which 7 component scores (0–3) are derived; the global score ranges from 0 to 21, with higher scores indicating poorer sleep quality. We used Chinese versions of the SDS, SAS, and PSQI that have been validated in Chinese populations (13, 19); additional Chinese-validation references can be cited as appropriate. Completion of the full questionnaire (KAP, SDS, SAS, PSQI) usually required 5–10 min.

Data were collected by trained teams in each hospital (one attending rheumatologist and two senior nurses, supervised by the head nurse) who received centralized training on eligibility assessment, consent, and questionnaire administration. Participants completed the questionnaire in a quiet area of the clinic or ward. Research staff checked each questionnaire immediately for completeness and logic. Questionnaire records were excluded if they had missing key sections, if the participant explicitly declined consent for research use, or if they contained malformed or internally implausible sleep-time entries that prevented valid PSQI scoring. Valid questionnaires were double-entered into a secure database.

### Sample size calculation

The sample size was calculated using the formula for cross-sectional studies: *n* = (*Z*_1−α/2_/δ)^2^ × *P* × (1−*P*) where *Z*_1−α/2_ = 1.96 for *α* = 0.05, *p* = 0.5 (maximum variability), and admissible error *δ* = 0.05. The minimum required sample was 384; allowing for approximately 20% non-response or invalid questionnaires, we targeted at least 480 participants.

### Statistical analysis

Statistical analyses were performed using SPSS 26.0 (IBM Corp., Armonk, NY, USA). Continuous variables were tested for normality (Shapiro–Wilk test, histograms, and Q–Q plots). Normally distributed variables were summarized as mean ± standard deviation (SD) and compared using Student’s *t*-test or one-way ANOVA; non-normally distributed variables were expressed as median (interquartile range, IQR) and compared using Mann–Whitney U or Kruskal–Wallis *H* tests. Categorical variables were described as frequencies (percentages) and compared using chi-square tests. Correlations among KAP scores and between KAP and SDS, SAS, and PSQI scores were examined. For approximately normally distributed variables, Pearson correlation coefficients were calculated; when assumptions were not met, we confirmed robustness with Spearman correlation (available on request). To identify factors associated with higher KAP levels, KAP scores were dichotomized according to the 70% cutoff and analyzed using univariable and multivariable logistic regression. Rather than data-driven univariable screening, an *a priori* confounder selection strategy was applied. Clinically predefined variables—including age, sex, education, monthly per capita household income, disease type indicators, and disease duration—were forced into all multivariable models. We checked model assumptions including adequate events per variable and multicollinearity (assessed via Variance Inflation Factors [VIF], with all VIFs < 5 indicating no significant collinearity). Adjusted odds ratios (ORs) and 95% confidence intervals (CIs) were reported.

Structural equation modeling (SEM) was used to explore the relationships among knowledge, attitude, and practice based on the KAP theoretical framework (14, 15). The model specified directional paths to evaluate whether knowledge is associated with attitude, attitude is associated with practice, and knowledge is directly associated with practice. SEM analyses were conducted using AMOS 26.0 with maximum likelihood estimation. Missing data were handled using an exclusion-based complete-case analysis approach, as records with missing key sections or invalid responses were excluded prior to scoring, and no missing-value imputation was performed. Model fit was evaluated using standard indices including *χ*^2^/df (CMIN/DF), root mean square error of approximation (RMSEA), incremental fit index (IFI), Tucker-Lewis index (TLI), and comparative fit index (CFI). To address potential information loss from dichotomization, sensitivity analyses were additionally performed using multiple linear regression models with the continuous KAP scores as the dependent variables, reporting unstandardized (B) and standardized (β) coefficients. To account for multiplicity and precision of estimation, effect sizes—including adjusted odds ratios (ORs), unstandardized (B) and standardized regression coefficients (beta), and correlation coefficients (r)—are presented alongside their corresponding 95% confidence intervals (CIs) rather than relying solely on *p*-values.

## Results

### Participant characteristics

A total of 916 questionnaire records were collected from the data-collection systems. Of these, 79 were excluded due to incomplete key questionnaire sections, 33 were excluded because research-use consent was not provided, and 132 were excluded because malformed or implausible sleep-time data prevented valid PSQI scoring. After sequential exclusion, 672 valid questionnaires remained in the final analytical dataset. The detailed participant flow is presented in Figure 1.

**Figure 1:**
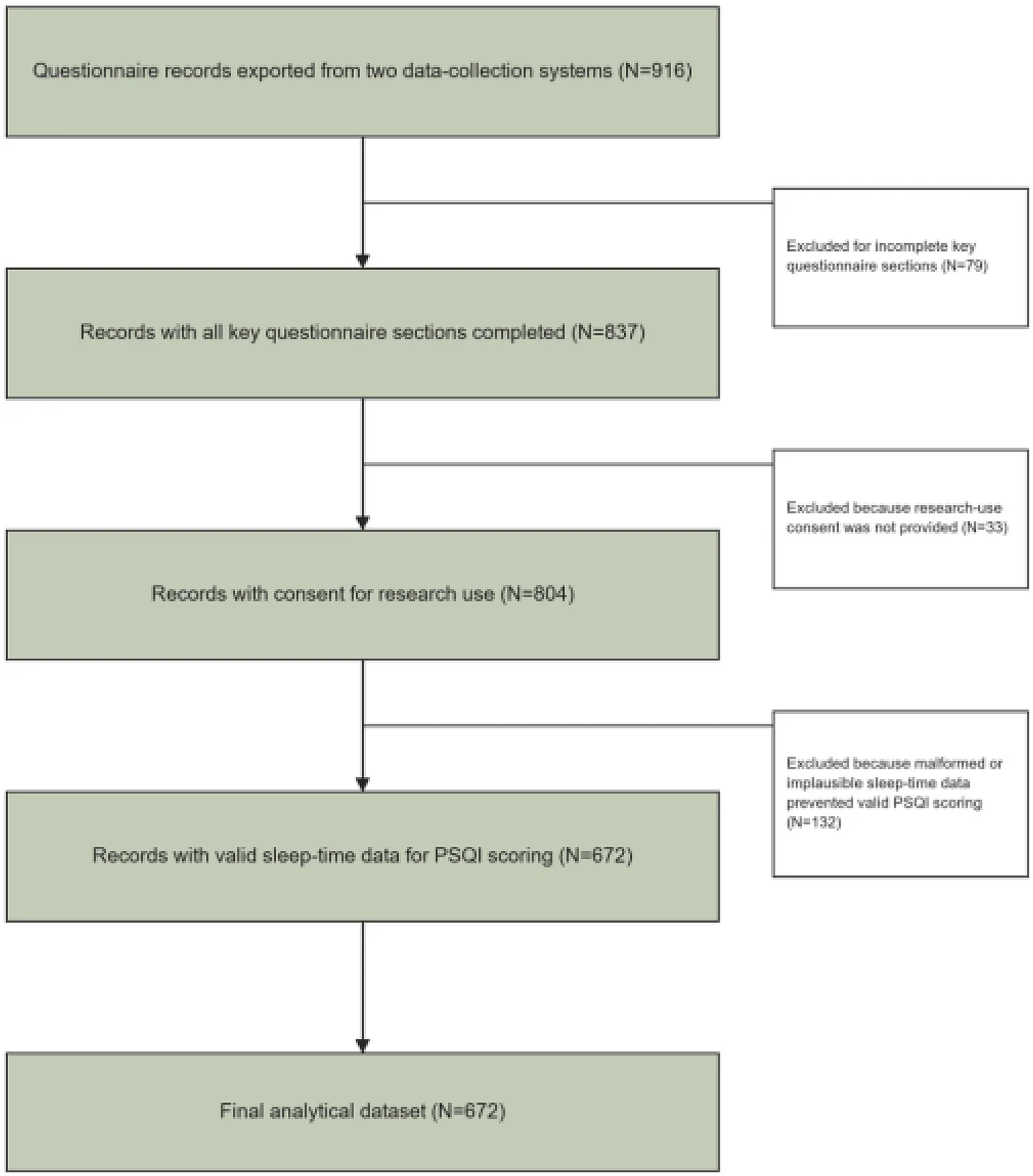
Participant flow.

Among the 672 patients with immune-mediated and inflammatory rheumatic diseases, 499 (74.26%) were female, with a mean age of 44.86 ± 15.54 years. Overall, 249 (37.05%) were employed, 252 (37.50%) had a monthly per capita household income < 2,000 yuan, and 514 (76.49%) were married. Regarding disease types, 162 (24.11%) had systemic lupus erythematosus, 121 (18.01%) had rheumatoid arthritis, 75 (11.16%) had Sjögren’s syndrome, and 63 (9.38%) had gout. Other rheumatologic conditions are shown in Table 1.

**Table 1:** Demographic characteristics and KAP scores (only variables with significant differences in ≥1 KAP domain).

Variable	Values	Knowledge (M ± SD)	*p*-value	Attitude (M ± SD)	*p*-value	Practice (M ± SD)	*p*-value
Total sample	*n* = 672 *n* = 672 *n* = 672	5.65 ± 4.81	–	23.03 ± 4.13	–	18.41 ± 4.18	–
Age (years)	44.86 ± 15.54	–	–	–	–	–	–
Residence			0.001		0.052		0.055
Non‑urban	256 (38.1)	4.89 ± 4.72		22.64 ± 4.36		18.12 ± 4.12	
Urban	416 (61.9)	6.12 ± 4.81		23.27 ± 3.96		18.58 ± 4.21	
Education			<0.001		<0.001		0.005
Primary school and below	206 (30.65)	4.10 ± 4.54		22.26 ± 3.92		17.85 ± 4.30	
Middle school	150 (22.32)	5.29 ± 4.68		22.58 ± 4.09		18.05 ± 4.12	
High school/technical secondary	236 (35.12)	6.99 ± 4.61		23.86 ± 3.93		19.08 ± 3.84	
Junior college/undergraduate+	80 (11.90)	6.39 ± 5.12		23.39 ± 4.80		18.49 ± 4.70	
Employment status			0.002		0.005		0.013
Employed	249 (37.05)	6.58 ± 4.90		23.65 ± 4.10		18.63 ± 4.25	
Unemployed	76 (11.31)	4.88 ± 4.06		22.09 ± 4.50		16.91 ± 4.15	
Retired	138 (20.54)	5.47 ± 4.84		22.66 ± 4.10		19.10 ± 4.24	
Housewife/househusband	124 (18.45)	4.74 ± 4.90		22.68 ± 3.73		18.22 ± 4.15	
Other	85 (12.65)	5.24 ± 4.62		23.15 ± 4.24		18.25 ± 3.66	
Monthly per capita household income (CNY)			<0.001		0.061		0.006
<2,000	252 (37.50)	4.57 ± 4.49		22.55 ± 4.17		17.85 ± 3.97	
2,000–5,000	233 (34.67)	6.39 ± 5.02		23.29 ± 4.08		18.51 ± 4.44	
>5,000	187 (27.83)	6.19 ± 4.72		23.35 ± 4.08		19.02 ± 4.04	
Marital status			0.102		0.004		0.783
Married	514 (76.49)	5.51 ± 4.82		22.80 ± 4.14		18.39 ± 4.11	
Other^*^	158 (23.51)	6.12 ± 4.76		23.78 ± 3.99		18.45 ± 4.40	
Drinking status			0.913		0.241		0.026
Never	499 (74.26)	5.72 ± 4.89		23.21 ± 4.06		18.64 ± 4.12	
Former drinker	135 (20.09)	5.47 ± 4.53		22.53 ± 4.28		17.75 ± 3.98	
Current drinker	38 (5.65)	5.47 ± 4.87		22.50 ± 4.39		17.66 ± 5.26	
Family history of rheumatic diseases			0.295		0.700		0.063
Yes	57 (8.48)	6.16 ± 5.29		23.65 ± 4.10		18.70 ± 4.06	
No	484 (72.02)	5.76 ± 4.83		22.97 ± 4.16		18.56 ± 4.22	
Not consulted/unclear	131 (19.49)	5.02 ± 4.51		22.99 ± 4.02		17.70 ± 4.03	
Family history of mental illness			0.218		0.618		0.060
Yes	11 (1.64)	8.73 ± 6.86		24.55 ± 5.22		16.91 ± 6.72	
No	525 (78.13)	5.64 ± 4.68		22.98 ± 4.20		18.57 ± 4.15	
Not consulted/unclear	136 (20.24)	5.45 ± 5.07		23.09 ± 3.75		17.90 ± 4.02	
Self-rating anxiety scale (SAS)	35.54 ± 6.87	–	–	–	–	–	–
Self-rating depression scale (SDS)	39.35 ± 10.15	–	–	–	–	–	–
Pittsburgh sleep quality index (PSQI)	7.08 ± 4.40	–	–	–	–	–	–

M ± SD, mean ± standard deviation. *p*-values from independent‑samples *t*-tests or one‑way ANOVA, as appropriate. ^*^“Other” marital status includes single, divorced, widowed, or separated.

The mean knowledge, attitude, and practice scores were 5.65 ± 4.81 (possible range: 0–18), 23.03 ± 4.13 (possible range: 6–30), and 18.41 ± 4.18 (possible range: 6–30), respectively. Knowledge scores differed significantly by education level (*p* < 0.001), work status (*p* = 0.002), residence (*p* = 0.001), and monthly per capita household income (*p* < 0.001). Attitude scores differed by education (*p* < 0.001), work status (*p* = 0.005), marital status (*p* = 0.004), and family history of rheumatic disease (*p* = 0.040). Practice scores differed by education (*p* = 0.005), work status (*p* = 0.013), monthly per capita household income (*p* = 0.006), and drinking habits (*p* = 0.026) (Table 1).

### Knowledge, attitude, and practice distributions

In the knowledge dimension, the items most frequently endorsed as “very well known” or “somewhat known” were K7 (“If someone suspects they have psychological problems or mental illnesses, they should seek consultation with a psychologist or a psychiatrist.”; 71.13%) and K3 (“Mental illnesses and psychological issues can be prevented, controlled, and improved through psychological interventions.”; 60.57%). By contrast, the items with the highest proportions of “unknown” responses were K6 (“Compared to the general population, family members of patients with immune-mediated and inflammatory rheumatic diseases are more in need of psychological interventions.”; 59.38%) and K5 (“Compared to the general population, patients with immune-mediated and inflammatory rheumatic diseases are more in need of psychological interventions.”; 58.93%) (Supplementary Table 2).

For the attitude items, response distributions were relatively similar across questions, with approximately 20% of patients choosing “strongly agree,” about 50% choosing “agree,” and around 25% selecting “neutral.” Specifically, 26.79% strongly agreed that psychological interventions are important for mental health (A1), 55.51% agreed that they would seek consultation from psychiatric or mental health services if they experienced symptoms such as anxiety or depression (A5), and 28.57% were neutral about accepting psychological interventions (A3) (Supplementary Table 3).

Regarding practice, 26.64% of respondents reported that they always and 41.37% that they often maintained an optimistic mindset (P6). Additionally, 41.22% always ensured sufficient sleep (P5), and 39.73% always ensured a regular diet and balanced nutrition (P4). In contrast, 37.95% reported that they only sometimes communicated with friends and relatives when they felt emotionally or physically unwell (P3). Notably, 41.90% never took the initiative to use psychological scales to assess their mental health status (P1), and 33.04% never actively learned about psychological interventions (P2) (Supplementary Table 4).

### Correlations among KAP, anxiety, depression, and sleep

Correlation analysis indicated significant positive correlations between knowledge and attitude (*r* = 0.388, *p* < 0.001), knowledge and practice (*r* = 0.416, *p* < 0.001), and attitude and practice (*r* = 0.342, *p* < 0.001). Anxiety symptoms and depressive symptoms were also positively correlated (*r* = 0.579, *p* < 0.001), as were anxiety symptoms and sleep disturbance (higher PSQI score; *r* = 0.390, *p* < 0.001) and depressive symptoms and sleep disturbance (*r* = 0.322, *p* < 0.001). In contrast, practice was negatively correlated with anxiety symptoms (*r* = −0.225, *p* < 0.001), depressive symptoms (*r* = −0.237, *p* < 0.001), and sleep disturbance (*r* = −0.181, *p* < 0.001) (Supplementary Table 5).

### Factors associated with knowledge, attitude, and practice

For multivariate logistic regression analyses, each KAP dimension was dichotomized using the predefined 70% cut-off (adequate vs. inadequate knowledge, positive vs. non-positive attitude, proactive vs. non-proactive practice). Odds ratios (ORs) therefore reflect the change in odds of being in the higher KAP category per one-unit increase in a continuous predictor (e.g., per 1-point increase in knowledge, attitude, SDS score, or per 1-year increase in age) or relative to a reference category for categorical predictors.

In the multivariable model for adequate knowledge, none of the predefined clinical variables showed a statistically significant independent association with adequate knowledge after adjusting for all forced-entry covariates (Table 2).

**Table 2:** Univariable and multivariable logistic regression for knowledge.

Knowledge dimension	Univariable OR (95% CI)	*p*	Multivariable OR (95% CI)	*p*
Age (per year)	0.994 (0.976–1.011)	0.477	1.000 (0.979–1.021)	0.992
Sex: female vs. male	1.363 (0.704–2.638)	0.358	1.638 (0.768–3.492)	0.202
Education: middle school vs. primary school and below	0.793 (0.400–1.570)	0.506	1.232 (0.491–3.092)	0.657
Education: high school/technical secondary vs. primary school and below	1.683 (0.979–2.896)	0.060	1.817 (0.761–4.336)	0.179
Education: junior college/undergraduate or above vs. primary school and below	1.394 (0.657–2.960)	0.387	1.603 (0.561–4.580)	0.379
Monthly per capita household income: 2,000–5,000 vs. < 2,000	1.605 (0.932–2.766)	0.088	1.973 (0.923–4.218)	0.080
Monthly per capita household income: >5,000 vs. < 2,000	1.292 (0.726–2.300)	0.383	1.653 (0.706–3.873)	0.247
Connective tissue disease: present vs. absent	0.519 (0.157–1.712)	0.281	0.553 (0.151–2.020)	0.370
Systemic lupus erythematosus: present vs. absent	1.099 (0.593–2.035)	0.765	1.042 (0.403–2.699)	0.932
Rheumatoid arthritis: present vs. absent	1.064 (0.535–2.117)	0.860	1.183 (0.471–2.970)	0.721
Ankylosing spondylitis: present vs. absent	1.178 (0.346–4.007)	0.793	1.513 (0.333–6.877)	0.592
Sjögren syndrome: present vs. absent	0.728 (0.281–1.882)	0.512	0.750 (0.257–2.195)	0.600
Inflammatory myopathy: present vs. absent	1.019 (0.302–3.440)	0.976	1.319 (0.348–5.000)	0.684
Systemic sclerosis: present vs. absent	1.157 (0.441–3.036)	0.767	1.310 (0.403–4.260)	0.653
Vasculitis: present vs. absent	0.985 (0.292–3.322)	0.981	1.297 (0.317–5.301)	0.718
Gout: present vs. absent	0.692 (0.242–1.978)	0.492	0.859 (0.241–3.060)	0.814
Undifferentiated/unclassified rheumatic diseases: present vs. absent	1.399 (0.741–2.641)	0.300	1.542 (0.599–3.969)	0.370
Disease duration (per year)	1.026 (0.989–1.065)	0.171	1.022 (0.980–1.066)	0.311

All covariates shown were forced into the multivariable model. Reference categories were male sex, primary school or below, monthly income <2,000 CNY, and absence of the indicated disease. Disease indicators were non-mutually exclusive. McFadden pseudo-*R*^2^ = 0.041; Hosmer-Lemeshow *χ*^2^ = 8.21, df = 8, *p* = 0.413.

In the forced-entry model for positive attitude, knowledge score and age were independently associated with the odds of having a positive attitude (Table 3). Each 1-point increase in knowledge score was associated with a 15.7% increase in the odds of a positive attitude (OR = 1.157, 95% CI: 1.103–1.212, *p* < 0.001). Each additional year of age was associated with a 1.8% decrease in the odds of a positive attitude (OR = 0.982 per year, 95% CI: 0.968–0.996, *p* = 0.011).

**Table 3:** Univariable and multivariable logistic regression for attitude.

Attitude dimension	Univariable OR (95% CI)	*p*	Multivariable OR (95% CI)	*p*
Knowledge score	1.165 (1.113–1.218)	<0.001	1.157 (1.103–1.212)	<0.001
Age (per year)	0.976 (0.965–0.987)	<0.001	0.982 (0.968–0.996)	0.011
Sex: female vs. male	1.403 (0.958–2.054)	0.082	1.430 (0.900–2.272)	0.130
Education: middle school vs. primary school and below	0.790 (0.529–1.180)	0.249	0.796 (0.473–1.341)	0.392
Education: high school/technical secondary vs. primary school and below	1.904 (1.294–2.801)	0.001	0.998 (0.563–1.771)	0.995
Education: junior college/undergraduate or above vs. primary school and below	0.872 (0.519–1.463)	0.603	0.667 (0.332–1.341)	0.255
Monthly per capita household income: 2,000–5,000 vs. < 2,000	0.899 (0.628–1.288)	0.562	0.897 (0.563–1.430)	0.648
Monthly per capita household income: >5,000 vs. < 2,000	1.497 (0.999–2.244)	0.051	1.264 (0.721–2.218)	0.413
Connective tissue disease: present vs. absent	1.007 (0.553–1.831)	0.983	1.450 (0.683–3.078)	0.334
Systemic lupus erythematosus: present vs. absent	1.280 (0.844–1.939)	0.245	1.414 (0.718–2.783)	0.316
Rheumatoid arthritis: present vs. absent	0.737 (0.480–1.133)	0.164	1.389 (0.723–2.668)	0.324
Ankylosing spondylitis: present vs. absent	1.453 (0.584–3.616)	0.422	2.189 (0.706–6.787)	0.175
Sjögren syndrome: present vs. absent	1.061 (0.611–1.842)	0.832	1.441 (0.718–2.889)	0.304
Inflammatory myopathy: present vs. absent	0.993 (0.454–2.172)	0.987	1.388 (0.521–3.697)	0.512
Systemic sclerosis: present vs. absent	0.766 (0.413–1.421)	0.398	1.194 (0.516–2.763)	0.678
Vasculitis: present vs. absent	1.035 (0.475–2.255)	0.930	1.817 (0.691–4.777)	0.226
Gout: present vs. absent	1.280 (0.688–2.381)	0.436	1.484 (0.648–3.400)	0.351
Undifferentiated/unclassified rheumatic diseases: present vs. absent	1.134 (0.725–1.774)	0.582	1.387 (0.699–2.752)	0.349
Disease duration (per year)	0.983 (0.959–1.009)	0.197	0.981 (0.953–1.010)	0.194

All covariates shown were forced into the multivariable model. Reference categories were male sex, primary school or below, monthly income <2,000 CNY, and absence of the indicated disease. Disease indicators were non-mutually exclusive. McFadden pseudo-*R*^2^ = 0.098; Hosmer-Lemeshow *χ*^2^ = 4.75, df = 8, *p* = 0.784.

In the forced-entry model for proactive practice, knowledge, attitude, and age were independently associated with the odds of proactive practice (Table 4). Each 1-point increase in knowledge score was associated with a 13.3% increase in the odds of proactive practice (OR = 1.133, 95% CI: 1.086–1.182, *p* < 0.001), and each 1-point increase in attitude score was associated with an 11.5% increase (OR = 1.115, 95% CI: 1.059–1.173, *p* < 0.001). Each additional year of age was associated with a 2.3% increase in the odds of proactive practice (OR = 1.023 per year, 95% CI: 1.008–1.037, *p* = 0.002).

**Table 4:** Univariable and multivariable logistic regression for practice.

Practice dimension	Univariable OR (95% CI)	*p*	Multivariable OR (95% CI)	*p*
Knowledge score	1.154 (1.112–1.198)	<0.001	1.133 (1.086–1.182)	<0.001
Attitude score	1.156 (1.104–1.210)	<0.001	1.115 (1.059–1.173)	<0.001
Age (per year)	1.012 (1.001–1.023)	0.027	1.023 (1.008–1.037)	0.002
Sex: female vs. male	1.067 (0.726–1.569)	0.741	1.173 (0.728–1.891)	0.512
Education: middle school vs. primary school and below	0.760 (0.501–1.152)	0.196	0.838 (0.475–1.478)	0.541
Education: high school/technical secondary vs. primary school and below	1.332 (0.943–1.882)	0.104	1.039 (0.595–1.812)	0.894
Education: junior college/undergraduate or above vs. primary school and below	1.214 (0.735–2.003)	0.448	1.068 (0.524–2.178)	0.856
Monthly per capita household income: 2,000–5,000 vs. < 2,000	1.183 (0.835–1.676)	0.345	1.149 (0.711–1.857)	0.572
Monthly per capita household income: >5,000 vs. < 2,000	1.294 (0.898–1.865)	0.167	1.381 (0.803–2.375)	0.244
Connective tissue disease: present vs. absent	0.946 (0.526–1.701)	0.853	1.132 (0.557–2.302)	0.731
Systemic lupus erythematosus: present vs. absent	0.635 (0.419–0.962)	0.032	0.914 (0.491–1.700)	0.776
Rheumatoid arthritis: present vs. absent	1.459 (0.962–2.214)	0.076	1.800 (0.993–3.262)	0.053
Ankylosing spondylitis: present vs. absent	1.057 (0.475–2.352)	0.891	1.383 (0.486–3.931)	0.543
Sjögren syndrome: present vs. absent	1.181 (0.704–1.980)	0.529	1.228 (0.645–2.337)	0.532
Inflammatory myopathy: present vs. absent	1.188 (0.567–2.487)	0.648	1.286 (0.527–3.141)	0.581
Systemic sclerosis: present vs. absent	1.378 (0.756–2.511)	0.296	1.945 (0.887–4.266)	0.097
Vasculitis: present vs. absent	0.718 (0.320–1.609)	0.420	0.910 (0.339–2.443)	0.851
Gout: present vs. absent	1.161 (0.664–2.031)	0.600	1.313 (0.619–2.781)	0.478
Undifferentiated/unclassified rheumatic diseases: present vs. absent	1.166 (0.767–1.772)	0.472	1.161 (0.617–2.185)	0.644
Disease duration (per year)	1.001 (0.975–1.027)	0.952	0.991 (0.961–1.023)	0.592

All covariates shown were forced into the multivariable model. Reference categories were male sex, primary school or below, monthly income <2,000 CNY, and absence of the indicated disease. Disease indicators were non-mutually exclusive. McFadden pseudo-*R*^2^ = 0.131; Hosmer-Lemeshow *χ*^2^ = 4.35, df = 8, *p* = 0.824.

### Structural equation modeling

The structural equation model examining the relationships among knowledge, attitude, and practice demonstrated good overall fit: CMIN/DF = 4.950, RMSEA = 0.077, IFI = 0.934, TLI = 0.922, and CFI = 0.934 (Supplementary Table 6). In the final model using standardized estimates with 95% bootstrap confidence intervals, knowledge showed a significant direct path to attitude (*β* = 0.369, 95% CI: 0.291–0.445, *p* = 0.001) and a direct path to practice (*β* = 0.518, 95% CI: 0.428–0.597, *p* = 0.001). Attitude also demonstrated a significant direct path to practice (*β* = 0.202, 95% CI: 0.126–0.279, *p* = 0.001). Furthermore, knowledge had a significant indirect association with practice mediated through attitude (*β* = 0.074, 95% CI: 0.047–0.109, *p* = 0.001) (Supplementary Table 7 and Figure 2).

**Figure 2:**
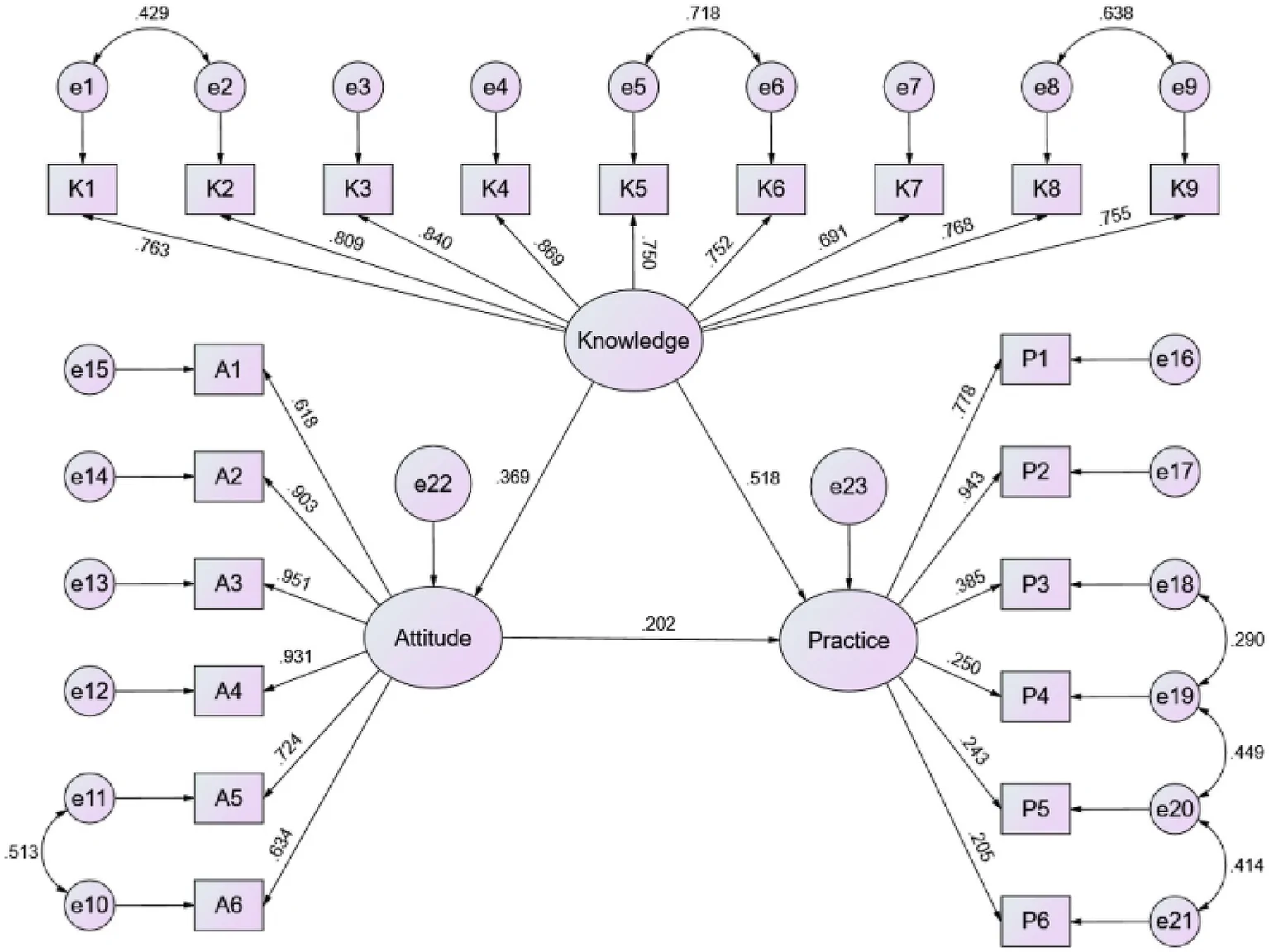
SEM path.

### Sensitivity analyses

Sensitivity analyses utilizing multiple linear regression with continuous KAP scores demonstrated highly consistent findings. In the continuous-score models, higher knowledge scores remained significantly associated with more positive attitudes (*β* = 0.365, *p* < 0.001). Furthermore, both higher knowledge scores (*β* = 0.352, *p* < 0.001) and higher attitude scores (*β* = 0.209, *p* < 0.001) were independently and significantly associated with higher practice scores (Supplementary Tables 8–10).

## Discussion

This multicenter cross-sectional study examined knowledge, attitudes, and practices regarding mental health and psychological interventions among patients with immune-mediated and inflammatory rheumatic diseases in China. We found that patients demonstrated limited knowledge, a generally positive attitude, but low engagement in mental health practices. Higher knowledge and more positive attitudes were strongly associated with more proactive practices, while age was also independently associated with practice. These findings support the need for targeted, culturally appropriate strategies to improve mental health literacy and translate positive attitudes into concrete behaviors in this population.

### Interpretation of main findings in relation to previous studies

Overall, patients’ knowledge about mental health and psychological interventions was inadequate, even though most acknowledged the importance of mental health care and expressed willingness to seek help if they experienced significant symptoms. This pattern—low knowledge, relatively favorable attitudes, and weak translation into action—aligns with findings from other chronic disease populations, where awareness of mental health does not necessarily lead to service use or self-management behaviors (16, 17). In our sample, patients were more likely to endorse general statements such as “psychological interventions are important” or “people with suspected mental illness should seek professional help”, but showed limited understanding of which patients are at higher risk (e.g., themselves and their family members) or of the specific nature and content of psychological interventions. Similar gaps in mental health literacy have been reported among patients with other chronic conditions and in the general Chinese population (20–23).

Sociodemographic factors were consistently related to KAP. Higher education was associated with better knowledge, more positive attitudes, and more proactive practices, underscoring the role of educational attainment in mental health literacy and help-seeking (22, 23). Higher monthly per capita household income was also associated with better knowledge and practice, which may reflect greater financial capacity to access care, as well as broader access to health information and digital resources (20, 21). Urban residence was related to higher knowledge and practice scores, likely due to better availability of mental health services and more frequent exposure to mental health–related information campaigns in urban areas. These patterns suggest that interventions should pay particular attention to patients with lower education, lower income, and those living in rural areas, who may face multiple barriers to understanding and accessing mental health support.

Regarding disease-specific factors, we included specific disease categories (such as systemic lupus erythematosus, rheumatoid arthritis, Sjögren’s syndrome, and gout) as covariates in our updated multivariable models to account for the varying clinical characteristics and psychological burdens across these conditions. Interestingly, after adjusting for socio-demographic factors, disease duration, and baseline knowledge/attitudes, specific disease types were generally not independent predictors of knowledge, attitude, or practice scores. This suggests that the identified gaps in mental health literacy and practice are relatively universal across the spectrum of immune-mediated and inflammatory rheumatic diseases in our cohort, underscoring the need for broad, cross-diagnostic psychological support strategies rather than strictly disease-specific educational interventions.

### Relationships among KAP, anxiety, depression, and sleep

The correlation and SEM analyses showed that knowledge, attitude, and practice are closely interrelated. Higher knowledge was associated with more positive attitudes and more proactive practices, and attitudes showed a strong direct path to practice. This pattern aligns with the classic KAP model (14, 15), in which knowledge is linked to beliefs and attitudes, which are in turn positively associated with behavior. Our SEM findings suggest that knowledge has an indirect statistical path to practice through its association with attitudes, while also showing a smaller direct path to practice. This highlights the need for programs that do more than simply provide information; they should also address patients’ beliefs, perceived benefits and risks, and self-efficacy regarding mental health care (24, 25).

We also found significant positive correlations among anxiety, depression, and poorer sleep quality, as well as negative associations between mental health practice and symptoms of anxiety and depression. Patients who engaged in more proactive mental health practices (e.g., maintaining a regular lifestyle, seeking social support) tended to report fewer symptoms, whereas those with more severe depressive symptoms were less likely to engage in proactive practice. Correlation analyses indicated that higher depression scores were associated with lower practice scores. This suggests a potentially bidirectional relationship: psychological distress may reduce motivation and energy to seek help or engage in healthy behaviors, while limited practice may, in turn, contribute to worsening symptoms (26, 27). Although our cross-sectional design cannot establish causality, the findings support the importance of early screening and support for depression and anxiety in rheumatology settings, both to address current suffering and to enable patients to benefit from educational and behavioral interventions.

### Determinants of knowledge, attitudes, and practices

Our updated forced-entry logistic regression analyses provided further insight into the independent determinants of KAP. After adjusting for predefined clinical covariates, socio-demographic factors that appeared significant in univariable trends were no longer independently significant for adequate knowledge. However, age demonstrated differential independent associations with attitudes and practice. In our models, older age was slightly associated with a lower likelihood of having a positive attitude but a higher likelihood of engaging in proactive practice. One possible explanation is that older patients, particularly those with long standing disease, may develop more stable routines and coping strategies (e.g., maintaining sleep hygiene, regular diet, or social support), even if their expressed attitudes toward mental health services are somewhat less favorable. Younger patients may endorse more positive attitudes or openness toward psychological interventions but may face competing work or family demands, or lack the experience and skills to translate these attitudes into sustained practice. Generational differences in awareness, stigma, and preferences for help seeking have been documented in studies of mental health in China and other countries (20, 21, 28). Tailoring interventions by age group—for example, offering more practical, behavior focused tools for younger patients, and more stigma reducing, attitude oriented interventions for older patients—may improve effectiveness.

### Knowledge, attitudes, and practices: implications for clinical care

Item level patterns across the KAP dimensions reveal specific targets for intervention. In the knowledge domain, many patients did not recognize that individuals with immune-mediated and inflammatory rheumatic diseases, and their family members, may have particularly high needs for psychological support. Limited understanding of who is “at risk” and what psychological interventions involve may partly explain why many patients never actively learned about psychologsssssssical interventions or used psychological scales to assess their mental health. Clarifying the goals, methods, and potential benefits of psychological interventions—such as psychoeducation, cognitive-behavioral strategies, stress management, and support groups—may reduce uncertainty and lower barriers to engagement (24, 25, 28, 29).

In the attitude domain, most patients agreed that psychological interventions are important and expressed willingness to seek professional help if they developed symptoms such as anxiety or depression. This provides a favorable foundation for integrating mental health services into rheumatology care. At the same time, some patients were hesitant about pharmacological treatment for mental health problems, which may reflect concerns about side effects, interactions with existing medications, or a preference for non-pharmacological approaches. Shared decision-making that presents a range of evidence-based options—including psychological therapies, lifestyle interventions, and, when appropriate, medication—may help align treatment plans with patients’ values and preferences (30, 31).

In terms of practice, patients reported relatively active behaviors in maintaining sleep, diet, and an optimistic mindset and in seeking support from family and friends. However, very few routinely used psychological assessment tools or actively sought information about psychological interventions. As reflected in our questionnaire, the “Practice” dimension encompasses a broad construct ranging from general mental health self-management to specific engagement in formal psychological interventions. While our Confirmatory Factor Analysis supported grouping these diverse behaviors into a single domain, the item-level differences suggest that although patients are already engaging in some general protective behaviors, they may not view structured mental health assessment or formal psychological services as part of routine care for their rheumatologic disease. Rheumatology teams could consider incorporating brief, validated mental health screening tools into regular visits and providing feedback to patients, thereby normalizing mental health assessment as a standard part of chronic disease management (32, 33). Simple, low-burden strategies—such as distributing brief educational leaflets, offering QR codes linking to reputable online resources, or embedding mental health information into patient education sessions—may further promote self-monitoring and timely help-seeking.

### Cultural context: stigma, access, and mental health literacy in China

These findings should also be interpreted in the context of mental health stigma, access barriers, and literacy challenges within China. Despite recent national initiatives to strengthen mental health services, many patients still perceive psychological problems as a source of shame or weakness, which can discourage disclosure and formal help-seeking. The term “psychological intervention” may be unfamiliar or associated mainly with severe mental illness in psychiatric hospitals, rather than with routine supportive care in general medical settings. Access to specialized mental health professionals is also uneven, with substantial urban–rural disparities and limited integration between rheumatology and mental health services in many hospitals. Low baseline mental health literacy, especially among individuals with lower education and income, may further limit recognition of symptoms and understanding of available treatments. Addressing these context-specific barriers—for example, by using non-stigmatizing language, integrating brief psychological support into rheumatology clinics, and increasing community-based education—will be essential for translating patients’ generally positive attitudes into effective, sustainable mental health practices.

### Limitations and implications for future research

This study has several limitations that should be considered when interpreting the findings. First, the cross-sectional design precludes causal inferences about the relationships among knowledge, attitudes, practices, and mental health symptoms. For example, although higher practice was associated with lower anxiety and depression, we cannot determine whether better practice leads to fewer symptoms, whether symptoms reduce patients’ capacity to engage in healthy behaviors, or both. Longitudinal studies are needed to clarify temporal and causal pathways. Second, the sampling strategy combined stratified selection of tertiary Grade A hospitals with convenience sampling of patients within each site. Although this approach allowed us to recruit a relatively large sample from multiple regions, it may have introduced selection bias. Patients attending large tertiary hospitals may differ from those treated in secondary or community settings in disease severity, socioeconomic status, access to information, and attitudes toward health care. As a result, the generalizability of our findings to all patients with immune-mediated and inflammatory rheumatic diseases in China, particularly those in rural or underresourced areas, may be limited. Third, all data were self-reported, including KAP responses and mental health measures, which are subject to recall bias, social desirability bias, and misunderstanding of questions. Although we excluded questionnaires with clear logical inconsistencies and used previously validated Chinese instruments for depression, anxiety, and sleep, some measurement error is inevitable. For the newly developed KAP questionnaire, while we have now established its internal consistency and construct validity using the full analytical sample, other psychometric properties—such as test–retest reliability and longitudinal responsiveness—could not be assessed due to the cross-sectional design without follow-up evaluations. Future research should strengthen the psychometric evaluation of the KAP instrument in diverse rheumatologic populations. Finally, although we included patients from multiple regions, the number of hospitals was limited, and the participating sites were all tertiary Grade A institutions. System-level factors such as local mental health resources, integration of psychosocial care into rheumatology services, and regional differences in health policy were not fully examined. Future multicenter studies including secondary and community hospitals, as well as qualitative research exploring patients’ and clinicians’ perspectives, could provide a more nuanced understanding of barriers and facilitators to mental health care in this population.

Despite these limitations, our study contributes new evidence on mental health–related KAP among patients with immune-mediated and inflammatory rheumatic diseases in China. The results highlight specific groups at higher risk of inadequate knowledge and practice, clarify the interrelationships between KAP and mental health symptoms, and underscore the importance of culturally sensitive, education-focused strategies within rheumatology services. Integrating systematic mental health assessment and tailored psychoeducation into routine care may help bridge the gap between patients’ generally positive attitudes and their low engagement in formal mental health practices.

## Conclusion

In conclusion, patients with immune-mediated and inflammatory rheumatic diseases demonstrated specific patterns in mental health knowledge, attitudes, and practices. Rather than establishing definitive intervention efficacy, these findings highlight key areas for future health-services research and underscore the hypothesis that targeted educational and psychological programs could potentially enhance patient engagement and wellbeing, which should be tested in rigorous longitudinal and interventional studies.

## Data Availability

The original contributions presented in the study are included in the article/Supplementary material, further inquiries can be directed to the corresponding authors.
