# Supplementary material for: Knowledge, attitude and practice of patients with immune-mediated and inflammatory rheumatic diseases towards mental health and psychological interventions
Source: Front Public Health. 2026 Oct 21;14:1959054. doi: 10.3389/fpubh.2026.1959054 (PMC13760827; doi:10.3389/fpubh.2026.1959054)
Supplement: Supplementary file 1 [file Data_sheet_1.docx]

**Supplementary Figure 1. CFA results**

**Supplementary Table 1. List of 6 institutions participating in this study**

| No, | Institution name |
| --- | --- |
| 1 | The First Hospital of China Medical University |
| 2 | Peking Union Medical College |
| 3 | Peking University People's Hospital |
| 4 | West China Hospital of Sichuan University |
| 5 | Tongji Hospital, Tongji Medical College, Huazhong University of Science and Technology |
| 6 | Shanxi Bethune Hospital, Shanxi Academy of Medical Sciences, Tongji Shanxi Hospital, Third Hospital of Shanxi Medical University |

**Supplementary Table 2. Distribution of knowledge dimension responses**

| **Knowledge** | **N (%)** | | |
| --- | --- | --- | --- |
|  | c. Unknown | b. Somewhat known | a. Very well known |
| K1 I understand what psychological interventions are.  Psychological interventions refer to a planned, step-by-step process under the guidance of psychological theories, systematically influencing the psychological activities, individual characteristics, or psychological issues of a specific target, aiming to bring about changes towards the anticipated goals. | 313(46.58) | 317(47.17) | 42(6.25) |
| K2 I know what aspects psychological interventions cover.  Psychological interventions include health promotion, preventive interventions, psychological counseling, and psychotherapy, among others. | 336(50) | 296(44.05) | 40(5.95) |
| K3 Mental illnesses and psychological issues can be prevented, controlled, and improved through psychological interventions. | 265(39.43) | 347(51.64) | 60(8.93) |
| K4 The scope of psychological interventions encompasses all populations, including the general population, high-risk groups, and individuals with psychological issues. | 347(51.64) | 268(39.88) | 57(8.48) |
| K5 Compared to the general population, patients with immune-mediated and inflammatory rheumatic diseases are more in need of psychological interventions. | 396(58.93) | 215(31.99) | 61(9.08) |
| K6 Compared to the general population, family members of patients with immune-mediated and inflammatory rheumatic diseases are more in need of psychological interventions. | 399(59.38) | 214(31.85) | 59(8.78) |
| K7 If someone suspects they have psychological problems or mental illnesses, they should seek consultation with a psychologist or a psychiatrist. | 194(28.87) | 334(49.7) | 144(21.43) |
| K8 Psychological interventions can positively impact and improve the negative emotions of patients with immune-mediated and inflammatory rheumatic diseases, increasing personal well-being. | 306(45.54) | 277(41.22) | 89(13.24) |
| K9 Psychological interventions can promote the self-recovery of patients with immune-mediated and inflammatory rheumatic diseases, thereby enhancing their overall quality of life. | 327(48.66) | 264(39.29) | 81(12.05) |

**Supplementary Table 3. Distribution of attitude dimension responses**

| **Attitude** | e.Strongly disagree | d.Disagree | c.Neutral | b.Agree | a.Strongly agree |
| --- | --- | --- | --- | --- | --- |
| A1 I believe that psychological interventions are very important for mental health. | 2(0.3) | 6(0.89) | 135(20.09) | 349(51.93) | 180(26.79) |
| A2 I am willing to undergo psychological assessment. | 4(0.6) | 34(5.06) | 158(23.51) | 345(51.34) | 131(19.49) |
| A3 I am willing to undergo psychological interventions (including psychological counseling, etc.). | 7(1.04) | 41(6.1) | 192(28.57) | 314(46.73) | 118(17.56) |
| A4 I am willing to receive health education related to psychological interventions. | 5(0.74) | 33(4.91) | 181(26.93) | 328(48.81) | 125(18.6) |
| A5 If symptoms such as anxiety or depression occur, I am willing to seek consultation from the psychiatric or mental health department. | 9(1.34) | 25(3.72) | 140(20.83) | 373(55.51) | 125(18.6) |
| A6 If symptoms such as anxiety or depression occur, I am willing to undergo treatment with psychiatric medications. | 9(1.34) | 40(5.95) | 174(25.89) | 334(49.7) | 115(17.11) |

**Supplementary Table 4. Distribution of practice dimension responses**

| **Practice** | e.Never | d.Rarely | c.Sometimes | b.Often | a.Always |
| --- | --- | --- | --- | --- | --- |
| P1 I will proactively use psychological assessments to evaluate my mental health status. | 282(41.96) | 185(27.53) | 150(22.32) | 31(4.61) | 24(3.57) |
| P2 I will proactively seek information about methods of psychological interventions. | 222(33.04) | 202(30.06) | 181(26.93) | 44(6.55) | 23(3.42) |
| P3 When my mood is not good or I feel physically unwell, I always actively communicate with family and friends. | 54(8.04) | 107(15.92) | 255(37.95) | 183(27.23) | 73(10.86) |
| P4 I always maintain a regular and balanced diet. | 21(3.13) | 44(6.55) | 208(30.95) | 277(41.22) | 122(18.15) |
| P5 I always ensure an adequate amount of sleep and maintain a regular daily routine. | 22(3.27) | 66(9.82) | 203(30.21) | 267(39.73) | 114(16.96) |
| P6 I always maintain an optimistic mindset. | 16(2.38) | 39(5.8) | 160(23.81) | 278(41.37) | 179(26.64) |

**Supplementary** **Table 5. Correlation analysis**

|  | Knowledge | Attitude | Practice | Anxiety | Depression | Sleep |
| --- | --- | --- | --- | --- | --- | --- |
| Knowledge | - |  |  |  |  |  |
| Attitude | 0.388(P<0.001) | - |  |  |  |  |
| Practice | 0.416(P<0.001) | 0.342(P<0.001) | - |  |  |  |
| Anxiety | -0.008(P=0.842) | 0.029(P=0.457) | -0.225(P<0.001) | - |  |  |
| Depression | -0.056(P=0.148) | -0.001(P=0.970) | -0.237(P<0.001) | 0.579(P<0.001) | - |  |
| Sleep | 0.020(P=0.610) | -0.038(P=0.324) | -0.181(P<0.001) | 0.390(P<0.001) | 0.322(P<0.001) | - |

**Supplementary Table 6. SEM model fit.**

| **Model I** | **Ref.** | **Measured results** |
| --- | --- | --- |
| **CMIN/DF** | 1-3 excellent,3-5 good | 4.950 |
| **RMSEA** | <0.08 good | 0.077 |
| **IFI** | >0.8 good | 0.934 |
| **TLI** | >0.8 good | 0.922 |
| **CFI** | >0.8 good | 0.934 |

**Supplementary Table 7. Results of SEM analysis**

| **Model paths** | **Standardized Total effects** | | **Standardized direct effects** | | **Standardized indirect effects** | |
| --- | --- | --- | --- | --- | --- | --- |
|  | **β (95%CI)** | **P** | **β (95%CI)** | **P** | **β (95%CI)** | **P** |
| Knowledge→Attitude | 0.369 (0.291,0.445) | 0.001 | 0.369 (0.291,0.445) | 0.001 |  |  |
| Knowledge→Practice | 0.592 (0.519,0.661) | 0.001 | 0.518 (0.428,0.597) | 0.001 |  |  |
| Attitude→Practice | 0.202 (0.126,0.279) | 0.001 | 0.202 (0.126,0.279) | 0.001 |  |  |
| Knowledge→Practice |  |  |  |  | 0.074 (0.047,0.109) | 0.001 |

|  |  |  | **Estimate** | **Standardized Estimate** | **S.E.** | **C.R.** | **P** |
| --- | --- | --- | --- | --- | --- | --- | --- |
| Attitude | <--- | Knowledge | 0.433 | 0.369 | 0.052 | 8.382 | <0.001 |
| Practice | <--- | Attitude | 0.311 | 0.202 | 0.060 | 5.167 | <0.001 |
| Practice | <--- | Knowledge | 0.935 | 0.518 | 0.084 | 11.140 | <0.001 |
| K1 | <--- | Knowledge | 1.000 | 0.763 |  |  |  |
| K2 | <--- | Knowledge | 1.060 | 0.809 | 0.036 | 29.149 | <0.001 |
| K3 | <--- | Knowledge | 1.138 | 0.840 | 0.050 | 22.954 | <0.001 |
| K4 | <--- | Knowledge | 1.214 | 0.869 | 0.051 | 23.868 | <0.001 |
| K5 | <--- | Knowledge | 1.068 | 0.750 | 0.053 | 20.088 | <0.001 |
| K6 | <--- | Knowledge | 1.063 | 0.752 | 0.053 | 20.147 | <0.001 |
| K7 | <--- | Knowledge | 1.056 | 0.691 | 0.058 | 18.314 | <0.001 |
| K8 | <--- | Knowledge | 1.158 | 0.768 | 0.056 | 20.666 | <0.001 |
| K9 | <--- | Knowledge | 1.126 | 0.755 | 0.056 | 20.250 | <0.001 |
| A6 | <--- | Attitude | 1.000 | 0.634 |  |  |  |
| A5 | <--- | Attitude | 1.075 | 0.724 | 0.047 | 23.075 | <0.001 |
| A4 | <--- | Attitude | 1.417 | 0.931 | 0.072 | 19.697 | <0.001 |
| A3 | <--- | Attitude | 1.503 | 0.951 | 0.075 | 19.963 | <0.001 |
| A2 | <--- | Attitude | 1.357 | 0.903 | 0.070 | 19.287 | <0.001 |
| A1 | <--- | Attitude | 0.832 | 0.618 | 0.058 | 14.364 | <0.001 |
| P1 | <--- | Practice | 1.000 | 0.778 |  |  |  |
| P2 | <--- | Practice | 1.209 | 0.943 | 0.060 | 20.076 | <0.001 |
| P3 | <--- | Practice | 0.498 | 0.385 | 0.051 | 9.753 | <0.001 |
| P4 | <--- | Practice | 0.279 | 0.250 | 0.045 | 6.253 | <0.001 |
| P5 | <--- | Practice | 0.268 | 0.243 | 0.044 | 6.059 | <0.001 |
| P6 | <--- | Practice | 0.237 | 0.205 | 0.046 | 5.114 | <0.001 |

**Supplementary Table 8. Multivariable linear regression sensitivity analysis for knowledge score**

| **Variable** | **Adjusted B (95% CI)** | **P** | **Standardized β** |
| --- | --- | --- | --- |
| Age (per year) | -0.015 (-0.042-0.012) | 0.277 | -0.048 |
| Sex: Female vs Male | 0.617 (-0.297-1.531) | 0.186 | 0.056 |
| Education: Middle school vs Primary school and below | 0.961 (-0.089-2.012) | 0.073 | 0.083 |
| Education: High school/technical secondary vs Primary school and below | 2.489 (1.424-3.554) | <0.001 | 0.247 |
| Education: Junior college/undergraduate or above vs Primary school and below | 1.726 (0.356-3.095) | 0.014 | 0.117 |
| Monthly per capita household income: 2,000-5,000 vs <2,000 | 1.057 (0.147-1.967) | 0.023 | 0.105 |
| Monthly per capita household income: >5,000 vs <2,000 | 0.427 (-0.617-1.471) | 0.423 | 0.040 |
| Connective tissue disease: present vs absent | -0.824 (-2.232-0.585) | 0.252 | -0.049 |
| Systemic lupus erythematosus: present vs absent | 0.177 (-1.046-1.401) | 0.776 | 0.016 |
| Rheumatoid arthritis: present vs absent | -0.131 (-1.340-1.078) | 0.832 | -0.010 |
| Ankylosing spondylitis: present vs absent | 0.073 (-1.956-2.102) | 0.944 | 0.003 |
| Sjögren syndrome: present vs absent | 0.158 (-1.123-1.438) | 0.810 | 0.010 |
| Inflammatory myopathy: present vs absent | 0.802 (-0.977-2.581) | 0.377 | 0.037 |
| Systemic sclerosis: present vs absent | 0.204 (-1.403-1.810) | 0.804 | 0.011 |
| Vasculitis: present vs absent | 0.277 (-1.542-2.095) | 0.766 | 0.013 |
| Gout: present vs absent | 0.681 (-0.816-2.178) | 0.373 | 0.041 |
| Pending investigation/unconfirmed: present vs absent | 1.033 (-0.234-2.299) | 0.110 | 0.084 |
| Disease duration (per year) | 0.013 (-0.047-0.073) | 0.671 | 0.018 |

R²=0.089; adjusted R²=0.064; F=3.54; P <0.001.

**Supplementary Table 9. Multivariable linear regression sensitivity analysis for attitude score**

| **Variable** | **Adjusted B (95% CI)** | **P** | **Standardized β** |
| --- | --- | --- | --- |
| Knowledge score | 0.312 (0.250-0.375) | <0.001 | 0.365 |
| Age (per year) | -0.022 (-0.044--0.001) | 0.045 | -0.085 |
| Sex: Female vs Male | 0.475 (-0.270-1.220) | 0.212 | 0.050 |
| Education: Middle school vs Primary school and below | -0.243 (-1.100-0.614) | 0.578 | -0.025 |
| Education: High school/technical secondary vs Primary school and below | 0.297 (-0.583-1.178) | 0.508 | 0.034 |
| Education: Junior college/undergraduate or above vs Primary school and below | 0.139 (-0.981-1.259) | 0.808 | 0.011 |
| Monthly per capita household income: 2,000-5,000 vs <2,000 | 0.120 (-0.623-0.864) | 0.751 | 0.014 |
| Monthly per capita household income: >5,000 vs <2,000 | -0.005 (-0.855-0.846) | 0.992 | -0.001 |
| Connective tissue disease: present vs absent | 0.324 (-0.824-1.472) | 0.581 | 0.023 |
| Systemic lupus erythematosus: present vs absent | 0.359 (-0.637-1.355) | 0.480 | 0.037 |
| Rheumatoid arthritis: present vs absent | -0.040 (-1.024-0.944) | 0.936 | -0.004 |
| Ankylosing spondylitis: present vs absent | 1.262 (-0.390-2.914) | 0.135 | 0.063 |
| Sjögren syndrome: present vs absent | 0.573 (-0.469-1.615) | 0.281 | 0.044 |
| Inflammatory myopathy: present vs absent | 0.688 (-0.761-2.137) | 0.353 | 0.037 |
| Systemic sclerosis: present vs absent | -0.229 (-1.537-1.079) | 0.732 | -0.015 |
| Vasculitis: present vs absent | 0.079 (-1.401-1.560) | 0.916 | 0.004 |
| Gout: present vs absent | 0.797 (-0.422-2.016) | 0.201 | 0.057 |
| Pending investigation/unconfirmed: present vs absent | 0.402 (-0.631-1.435) | 0.446 | 0.038 |
| Disease duration (per year) | -0.016 (-0.065-0.033) | 0.532 | -0.025 |

N=668; R²=0.179; adjusted R²=0.155; F=7.45; P <0.001.

**Supplementary Table 10. Multivariable linear regression sensitivity analysis for practice score**

| **Variable** | **Adjusted B (95% CI)** | **P** | **Standardized β** |
| --- | --- | --- | --- |
| Knowledge score | 0.304 (0.239-0.368) | <0.001 | 0.352 |
| Attitude score | 0.211 (0.137-0.285) | <0.001 | 0.209 |
| Age (per year) | 0.038 (0.017-0.060) | <0.001 | 0.144 |
| Sex: Female vs Male | 0.512 (-0.208-1.232) | 0.164 | 0.054 |
| Education: Middle school vs Primary school and below | -0.062 (-0.889-0.765) | 0.883 | -0.006 |
| Education: High school/technical secondary vs Primary school and below | 0.172 (-0.678-1.022) | 0.691 | 0.020 |
| Education: Junior college/undergraduate or above vs Primary school and below | -0.176 (-1.256-0.905) | 0.750 | -0.014 |
| Monthly per capita household income: 2,000-5,000 vs <2,000 | 0.072 (-0.646-0.789) | 0.844 | 0.008 |
| Monthly per capita household income: >5,000 vs <2,000 | 0.718 (-0.102-1.538) | 0.087 | 0.078 |
| Connective tissue disease: present vs absent | 0.548 (-0.559-1.656) | 0.332 | 0.038 |
| Systemic lupus erythematosus: present vs absent | 0.085 (-0.876-1.047) | 0.862 | 0.009 |
| Rheumatoid arthritis: present vs absent | 0.340 (-0.609-1.289) | 0.483 | 0.032 |
| Ankylosing spondylitis: present vs absent | 1.127 (-0.469-2.723) | 0.167 | 0.056 |
| Sjögren syndrome: present vs absent | 0.800 (-0.206-1.806) | 0.120 | 0.061 |
| Inflammatory myopathy: present vs absent | 0.697 (-0.701-2.096) | 0.329 | 0.037 |
| Systemic sclerosis: present vs absent | 0.327 (-0.935-1.588) | 0.612 | 0.021 |
| Vasculitis: present vs absent | -0.059 (-1.488-1.369) | 0.935 | -0.003 |
| Gout: present vs absent | 0.566 (-0.611-1.743) | 0.347 | 0.040 |
| Pending investigation/unconfirmed: present vs absent | 0.457 (-0.540-1.453) | 0.370 | 0.043 |
| Disease duration (per year) | 0.009 (-0.038-0.057) | 0.701 | 0.014 |

R²=0.250; adjusted R²=0.227; F=10.77; P <0.001.
