# Supplementary material for: Knowledge, attitude and practice of patients with immune-mediated and inflammatory rheumatic diseases towards mental health and psychological interventions
Source: Front Public Health. 2026 Oct 21;14:1959054. doi: 10.3389/fpubh.2026.1959054 (PMC13760827; doi:10.3389/fpubh.2026.1959054)
Supplement: Supplementary file 2 [file Supplementary_file_1.docx]

**Knowledge, attitude and practice of patients with autoimmune rheumatologic diseases towards mental health and psychological interventions: A multicenter cross-sectional study**

| **Part I Basic Information** | | |
| --- | --- | --- |
| **1.Your gender:** | a.Male | b.Female |
| **2.Your age:** | | |
| **3.Your residence:** | a.Rural  b.Urban | |
| **4.Your education:** | a.Primary school and below  b.Middle school  c.High school/Technical secondary school  d.Junior college/Undergraduate  e.Master and above | |
| **5.Your occupation type/employment status:** | a.Employed  b.Unemployed  c.Retired  d.Student  e.Housewife/Househusband  f.Other (please fill in) | |
| **6.In the past year, your average monthly income was (including in-kind income, rental income, etc.):______Yuan** | a.<2000  b.2000-5000  c.5000-10000  d.10000-20000  e.>20000 | |
| **7.Your marital status:** | a.Unmarried  b.Married  c.Divorced  d.Widowed | |
| **8.Do you have a smoking habit?** | a.Never smoked  b.Used to smoke, but quit  c.Still smoking | |
| **9.Do you have a drinking habit?** | a.Never drunk  b.Used to drink, but quit  c.Still drinking | |
| **10.The rheumatic diseases you suffer from is (multiple choice):** | a.Connective tissue diseases  b.Systemic lupus erythematosus  c.Rheumatoid arthritis  d.Ankylosing spondylitis  e.Sjogren syndrome  f.Inflammatory myopathy  g.Systemic sclerosis  h.Vasculitis  i.Gout  j.Pending investigation, unconfirmed  k.Other (please fill in) | |
| **11.Has your family member ever been diagnosed with rheumatic diseases?** | a.Yes  b.No  c.Not consulted, unclear | |
| **12.Has your family member ever been diagnosed with mental illness (including depression, bipolar disorder, schizophrenia, anxiety disorder, obsessive-compulsive disorder, etc.)?** | a.Yes  b.No  c.Not consulted, unclear | |

| **Part II Knowledge of psychological interventions** | | | |  |
| --- | --- | --- | --- | --- |
| **1. I understand what psychological interventions are.**  Psychological interventions refer to a planned, step-by-step process under the guidance of psychological theories, systematically influencing the psychological activities, individual characteristics, or psychological issues of a specific target, aiming to bring about changes towards the anticipated goals. | a.Very well known | b.Somewhat known | c.Unknown | |
| **2. I know what aspects psychological interventions cover.**  Psychological interventions include health promotion, preventive interventions, psychological counseling, and psychotherapy, among others. | a.Very well known | b.Somewhat known | c.Unknown | |
| **3. Mental illnesses and psychological issues can be prevented, controlled, and improved through psychological interventions.** | a.Very well known | b.Somewhat known | c.Unknown | |
| **4. The scope of psychological interventions encompasses all populations, including the general population, high-risk groups, and individuals with psychological issues.** | a.Very well known | b.Somewhat known | c.Unknown | |
| **5. Compared to the general population, rheumatoid patients are more in need of psychological interventions.** | a.Very well known | b.Somewhat known | c.Unknown | |
| **6. Compared to the general population, family members of rheumatoid patients are more in need of psychological interventions.** | a.Very well known | b.Somewhat known | c.Unknown | |
| **7. If someone suspects they have psychological problems or mental illnesses, they should seek consultation with a psychologist or a psychiatrist.** | a.Very well known | b.Somewhat known | c.Unknown | |
| **8. Psychological interventions can positively impact and improve the negative emotions of rheumatoid patients, increasing personal well-being.** | a.Very well known | b.Somewhat known | c.Unknown | |
| **9. Psychological interventions can promote the self-recovery of rheumatoid patients, thereby enhancing their overall quality of life.** | a.Very well known | b.Somewhat known | c.Unknown | |

| **Part III Attitude to psychological interventions** | | | | | |
| --- | --- | --- | --- | --- | --- |
| **1. I believe that psychological interventions are very important for mental health.** | a.Strongly agree | b.Agree | c.Neutral | d.Disagree | e.Strongly disagree |
| **2. I am willing to undergo psychological assessment.** | a.Strongly agree | b.Agree | c.Neutral | d.Disagree | e.Strongly disagree |
| **3. I am willing to undergo psychological interventions (including psychological counseling, etc.).** | a.Strongly agree | b.Agree | c.Neutral | d.Disagree | e.Strongly disagree |
| **4. I am willing to receive health education related to psychological interventions.** | a.Strongly agree | b.Agree | c.Neutral | d.Disagree | e.Strongly disagree |
| **5. If symptoms such as anxiety or depression occur, I am willing to seek consultation from the psychiatric or mental health department.** | a.Strongly agree | b.Agree | c.Neutral | d.Disagree | e.Strongly disagree |
| **6. If symptoms such as anxiety or depression occur, I am willing to undergo treatment with psychiatric medications.** | a.Strongly agree | b.Agree | c.Neutral | d.Disagree | e.Strongly disagree |

| **Part IV Practice on psychological health and interventions** | |
| --- | --- |
| **1. I will proactively use psychological assessments to evaluate my mental health status.** | a.Always b.Often c.Sometimes d.Rarely e.Never |
| **2. I will proactively seek information about methods of psychological interventions.** | a.Always b.Often c.Sometimes d.Rarely e.Never |
| **3. When my mood is not good or I feel physically unwell, I always actively communicate with family and friends.** | a.Always b.Often c.Sometimes d.Rarely e.Never |
| **4. I always maintain a regular and balanced diet.** | a.Always b.Often c.Sometimes d.Rarely e.Never |
| **5. I always ensure an adequate amount of sleep and maintain a regular daily routine.** | a.Always b.Often c.Sometimes d.Rarely e.Never |
| **6. I always maintain an optimistic mindset.** | a.Always b.Often c.Sometimes d.Rarely e.Never |

**Part V Relevant Psychological Scales**

**Zung Self-Rating Anxiety Scale (SAS)**

Guideline: Please read the following carefully, select and tick the answers according to your actual feelings in the last 1 week, and calculate the total score after completing all the questions.

| No. | Item | A Little Of The Time | Some Of The Time | Good Part Of The Time | Most Of The Time |
| --- | --- | --- | --- | --- | --- |
| 1 | I feel more nervous and anxious than usual. | ① | ② | ③ | ④ |
| 2 | I feel afraid for no reason at all. | ① | ② | ③ | ④ |
| 3 | I get upset easily or feel panicky. | ① | ② | ③ | ④ |
| 4 | I feel like I’m falling apart and going to pieces. | ① | ② | ③ | ④ |
| 5* | I feel that everything is all right and nothing bad will happen. | ① | ② | ③ | ④ |
| 6 | My arms and legs shake and tremble. | ① | ② | ③ | ④ |
| 7 | I am bothered by headaches neck and back pain. | ① | ② | ③ | ④ |
| 8 | I feel weak and get tired easily. | ① | ② | ③ | ④ |
| 9* | I feel calm and can sit still easily. | ① | ② | ③ | ④ |
| 10 | I can feel my heart beating fast. | ① | ② | ③ | ④ |
| 11 | I am bothered by dizzy spells. | ① | ② | ③ | ④ |
| 12 | I have fainting spells or feel like it. | ① | ② | ③ | ④ |
| 13* | I can breathe in and out easily. | ① | ② | ③ | ④ |
| 14 | I get numbness and tingling in my fingers and toes. | ① | ② | ③ | ④ |
| 15 | I am bothered by stomach aches or indigestion. | ① | ② | ③ | ④ |
| 16 | I have to empty my bladder often. | ① | ② | ③ | ④ |
| 17* | My hands are usually dry and warm. | ① | ② | ③ | ④ |
| 18 | My face gets hot and blushes. | ① | ② | ③ | ④ |
| 19* | I fall asleep easily and get a good night’s rest. | ① | ② | ③ | ④ |
| 20 | I have nightmares. | ① | ② | ③ |  |

**Zung Self-Depression Scale (SDS)**

Guideline: There are 20 items below, please read each item carefully to understand the meaning. After each item, there are four choices, which indicate: A None Or Little Of The Time (no more than 1 day in the past week); B Some Of The Time (1-2 days in the past week); C Good Part Of The Time (3-4 days in the past week); D Most Of The Time (5-7 days in the past week).

| No. | Item | A None Or Little Of The Time (no more than 1 day in the past week) | B Some Of The Time (1-2 days in the past week) | C Good Part Of The Time (3-4 days in the past week) | D Most Of The Time (5-7 days in the past week) |
| --- | --- | --- | --- | --- | --- |
| 1 | I feel down hearted and blue. | A | B | C | D |
| 2 | Morning is when I feel the best. | A | B | C | D |
| 3 | I have crying spells or feel like it. | A | B | C | D |
| 4 | I have trouble sleeping at night. | A | B | C | D |
| 5 | I eat as much as I used to. | A | B | C | D |
| 6 | I still enjoy sex. | A | B | C | D |
| 7 | I notice that I am losing weight. | A | B | C | D |
| 8 | I have trouble with constipation. | A | B | C | D |
| 9 | My heart beats faster than usual. | A | B | C | D |
| 10 | I get tired for no reason. | A | B | C | D |
| 11 | My mind is as clear as it used to be. | A | B | C | D |
| 12 | I find it easy to do the things I used to. | A | B | C | D |
| 13 | I am restless and can’t keep still. | A | B | C | D |
| 14 | I feel hopeful about the future. | A | B | C | D |
| 15 | I am more irritable than usual. | A | B | C | D |
| 16 | I find it easy to make decisions. | A | B | C | D |
| 17 | I feel that I am useful and needed. | A | B | C | D |
| 18 | My life is pretty full. | A | B | C | D |
| 19 | I feel that others would be better off if I were dead. | A | B | C | D |
| 20 | I still enjoy the things I used to do. | A | B | C | D |
|  |  |  |  |  |  |

**V. Scale Content**

**Please rate based on how you have felt in the past week. The numbers are ordered as follows: Never, Sometimes.**

1. **I feel depressed and gloomy. 1 2 3 4**

***2. I feel my mood is best in the morning. 4 3 2 1**

**3. I feel like crying or want to cry. 1 2 3 4**

**4. I have trouble sleeping at night. 1 2 3 4**

***5. I eat as much as usual. 4 3 2 1**

***6. My sexual function is normal. 4 3 2 1**

7. **I feel I have lost weight. 1 2 3 4**
8. **I am bothered by constipation. 1 2 3 4**
9. **My heart beats faster than usual. 1 2 3 4**
10. **I feel fatigued for no reason. 1 2 3 4**

***11. My mind is as clear as usual. 4 3 2 1**

***12. I do not find it difficult to do things as usual. 4 3 2 1**

13. **I feel restless and find it hard to stay calm. 1 2 3 4**

***14. I feel hopeful about the future. 4 3 2 1**

**15. I get irritated more easily than usual. 1 2 3 4**

***16. I find it easy to make decisions. 4 3 2 1**

***17. I feel that I am useful and indispensable. 4 3 2 1**

***18. My life is meaningful. 4 3 2 1**

19. **If I died, others would live better. 1 2 3 4**

***20. I still love the things I usually love. 4 3 2 1**

**Result Analysis:**

**The score is based on the total score. The raw score is obtained by adding up the scores of the 20 items. The standard score is the integer part of the raw score multiplied by 1.25. The normal upper limit of the total raw score is 41, and the standard total score is 53. The total score of the Self-Rating Depression Scale (SDS) is the sum of the scores for each item. Items 2, 5, 6, 11, 12, 14, 16, 17, 18, and 20 are reverse-scored. The results of the SDS are determined based on the standard score: A standard score less than 50 indicates no depression. A standard score between 50 and 59 indicates mild to moderate depression. A standard score between 60 and 69 indicates moderate to severe depression. A standard score of 70 or above indicates severe depression. The severity of depression is calculated as the total score of all items divided by 80. Results are interpreted as follows: Below 0.5: No depression; 0.5–0.59: Mild to moderate depression; 0.6–0.69: Moderate to severe depression; Above 0.7: Severe depression. This is for reference only.**

**Pittsburgh sleep quality index, PSQI**

Guideline: The following questions are about your sleep in the past month. Please select or fill in the answer that best matches your actual situation in the past month. Please answer the following questions!

| 1 | During the past month, what time have you usually gone to bed at night? ___________________ | | | | |
| --- | --- | --- | --- | --- | --- |
| 2 | During the past month, how long (in minutes) has it usually taken you to fall asleep each night? __________ | | | | |
| 3 | During the past month, what time have you usually gotten up in the morning? ___________________ | | | | |
| 4 | During the past month, how many hours of actual sleep did you get at night? (This may be different than the number of hours you spent in bed.) ___________________ | | | | |
| Please choose 1 answer to the following questions that best suits you. | | | | | |
| 5 | During the past month, how often have you had trouble sleeping because you… | | | | |
|  | a. Cannot get to sleep within 30 minutes | (1) Not during the past month | (2) Less than once a week | (3) Once or twice a week | (4) Three or more times a week |
|  | b. Wake up in the middle of the night or early morning | (1) Not during the past month | (2) Less than once a week | (3) Once or twice a week | (4) Three or more times a week |
|  | c. Have to get up to use the bathroom | (1) Not during the past month | (2) Less than once a week | (3) Once or twice a week | (4) Three or more times a week |
|  | d. Cannot breathe comfortably | (1) Not during the past month | (2) Less than once a week | (3) Once or twice a week | (4) Three or more times a week |
|  | e. Cough or snore loudly | (1) Not during the past month | (2) Less than once a week | (3) Once or twice a week | (4) Three or more times a week |
|  | f. Feel too cold | (1) Not during the past month | (2) Less than once a week | (3) Once or twice a week | (4) Three or more times a week |
|  | g. Feel too hot | (1) Not during the past month | (2) Less than once a week | (3) Once or twice a week | (4) Three or more times a week |
|  | h. Have bad dreams | (1) Not during the past month | (2) Less than once a week | (3) Once or twice a week | (4) Three or more times a week |
|  | i. Have pain | (1) Not during the past month | (2) Less than once a week | (3) Once or twice a week | (4) Three or more times a week |
|  | j. Other reason(s) | (1) Not during the past month | (2) Less than once a week | (3) Once or twice a week | (4) Three or more times a week |
|  | If yes, please describe: | | | | |
| 6 | During the past month, how would you rate your sleep quality overall? | (1) Very good | (2) Fairly good | (3) Fairly bad | (4) Very bad |
| 7 | During the past month, how often have you taken medicine to help you sleep? | (1) Not during the past month | (2) Less than once a week | (3) Once or twice a week | (4) Three or more times a week |
| 8 | During the past month, how often have you had trouble staying awake? | (1) Not during the past month | (2) Less than once a week | (3) Once or twice a week | (4) Three or more times a week |
| 9 | During the past month, how much of a problem has it been for you to keep up enough enthusiasm to get things done? | (1) No problem at all | (2) Only a very slight problem | (3) Somewhat of a problem | (4) A very big problem |
